# An Integrated Bile Acids Profile Determination by UHPLC-MS/MS to Identify the Effect of Bile Acids Supplement in High Plant Protein Diet on Common Carp (*Cyprinus carpio*)

**DOI:** 10.3390/foods10102465

**Published:** 2021-10-15

**Authors:** Xian Wei, Ting Yao, Fatou Ndoye Fall, Min Xue, Xiaofang Liang, Jie Wang, Wenlong Du, Xu Gu

**Affiliations:** 1 National Aquafeed Safety Assessment Center, Institute of Feed Research of CAAS, Beijing 100081, China; 82101195209@caas.cn (X.W.); yaoting0515@126.com (T.Y.); fatoundoye11@gmail.com (F.N.F.); xuemin@caas.cn (M.X.); liangxiaofang01@caas.cn (X.L.); wangjie03@caas.cn (J.W.); duwenlong0302@163.com (W.D.); 2 Beijing Institute of Feed Control, Beijing 100107, China

**Keywords:** bile acids, common carp, high plant protein diet, hepatopancreas, glycogen accumulation, UHPLC-MS/MS

## Abstract

Bile acids (BAs) have considerable importance in the metabolism of glycolipid and cholesterol. The purpose of the present study is to clarify the effects of bile acids supplementary in a high plant protein diet for the common carp BA profiles and hepatopancreas and intestine health. An 11-week feeding trial was conducted with high plant protein diet (18% soybean meal and 18% cottonseed protein concentrated) (HP) and HP added 600 mg/kg BAs (HP+BAs) for common carp, and then, the UHPLC-MS/MS technology was used to analyze the BAs in the bile and plasma of two groups. HP could induce vacuolation of hepatocytes and accumulation of glycogen in the common carp, while these phenotypes were significantly improved in the HP+BAs group. In addition, the BA profile of the HP group and HP+BAs group are described in detail, for the common carp bile with treatment by exogenous BAs, TCA, CA, T*β*MCA, and T*ω*MCA were the main components. Furthermore, in the HP+BAs group plasma, CDCA, CA, LCA, and GCDCA increased significantly; they could activate TGR5, and the activation of hepatopancreas TGR5 might regulate glucose metabolism to relieve hepatopancreas glycogen accumulation. This study proved that BAs supplemented to plant protein diet could relieve the common carp hepatopancreas glycogen accumulation by changing the BAs’ profile, thereby promoting its healthy growth, which has important guiding significance for the promotion of aquaculture development and makes an important contribution to expanding the strategic space of food security.

## 1. Introduction

Bile acids (BAs) are a series of amphipathic molecules that are synthesized in the liver from cholesterol and stored in the gallbladder [1]. Most of the BAs are conjugated with taurine or glycine in liver [2], and then hydrolyzed, dehydroxylated, and deconjugated in the gut [3,4]. BAs are secreted into the duodenum to promote lipid digestion and absorption in the small intestine and then are reabsorbed in the ileum by the liver via BAs transporters and the portal vein, which is defined as metabolism enterohepatic circulation [5]. BAs have been known to accelerate the digestion and absorption of lipids in the gut [6], and to regulate cholesterol homeostasis [7]. Moreover, in recent years, scientists have found that BAs also act as various signal receptors to participate in the regulations of homeostasis of glucose and energy metabolism [8], as well as in signaling pathways [9,10]. LCA (Lithocholic acid) and DCA (Deoxycholic acid) activate TGR5 (G protein coupled bile acid receptor 1) to regulate glucose metabolism and anti-inflammatory response [11,12]. T*β*MCA (Tauro-*β*-muricholic acid) and CDCA (Chenodeoxycholic acid) act together on FXR (Farnesoid X receptor) to regulate BAs’ synthesis and glycolipid metabolism [13,14]. These new findings of BAs’ functions helped to solve various diseases caused by metabolic disorders.

In order to meet the needs of environmental protection and reduce the cost of feed, plant protein is increasingly widely used in aquatic feed [15]. However, the application of plant protein could induce intestine damage and interfere with BAs’ metabolism, which results in hepatic lesions and disrupts the body’s overall metabolism, and finally, reduces fish growth performance remarkably as well as the efficiency of breeding. [16,17]. Bile acids were widely used in aquaculture of China, and they have a positive effect on fish growth performance, nutrient digestibility, and immunity [18,19]. However, not all BAs have a positive effect; some BAs would bring negative effects; for example, both TCA (Taurocholic acid) and bovine bile salt supplementation in a low fishmeal diet to the Atlantic salmon could cause slight or moderate inflammation of the distal intestine [20]. At present, in mammals, the profile of BAs generated a lot of results and interesting discoveries [21,22], while there were few reports that concentrated on BAs in fish, since most of them focused on the roles of BAs in fish pheromone systems and the identification of some new BAs in saltwater fish [23,24,25,26]. In general, BAs in fish have undergone fewer studies, which are relatively one-sided [27,28,29]. Previous studies by our team suggested that high plant protein induced common carp liver injury, which BA supplements could help to alleviate [30], but the questions of which BA played the leading role or how they affect liver health remain unanswered and BA profiles of fish are still unknown.

Common carp (*Cyprinus carpio*) [31] is a kind of omnivorous fish, an important economical freshwater fish around the world. It is estimated by the FAO that by 2030, freshwater species such as carp, catfish (including pangasius), and tilapia will account for about 62% of the global aquaculture production. The common carp needs a certain amount of animal protein, and a high proportion of plant protein may cause intestinal and liver diseases, thereby reducing the benefit of breeding. Therefore, the present study combined the UHPLC-MS/MS technology to explore the BA profile of the common carp comprehensively and the effect of BA supplement in high plant protein feed on the common carp’s BA profile and hepatopancreas health.

## 2. Materials and Methods

During the feeding period, the experimental fishes were maintained in compliance with the Laboratory Animal Welfare Guidelines of China (General Administration of Quality Supervision, Inspection and Quarantine of the People’s Republic of China, Standardization Administration of China, GB/T 35,892–2018).

### 2.1. Chemicals and Reagents

Reference standards of unconjugated and conjugated BAs (list in Table 1) including cholic acid-d4 (CA-d4), chenodeoxycholic acid-d4 (CDCA-d4), lithocholic acid-d4 (LCA-d4), and glycocholic acid -d4 (GCA-d4) were purchased from Steraloids Inc. (Newport, RI, USA). Taurocholic acid-d5 sodium salt (TCA-d5), tauro-*β*-muricholic acid-d4 sodium salt (T*β*MCA-d4), and tauroursodeoxycholic acid-d5 (TUDCA-d5) were obtained from Toronto Research Chemicals (North York, Ontario, Canada). *β*-muricholic acid-d5 *(β*MCA-d5) was bought from IsoSciences (Ambler, PA, USA). Seven deuterium-labeled BAs containing deoxycholic acid-d4 (DCA-d4), glycolithocholic acid-d4 (GLCA-d4), glycoursodeoxycholic acid-d4 (GUDCA-d4), taurodeoxycholic acid-d4 sodium salt (TDCA-d4), glycochenodeoxycholic acid-d4 (GCDCA-d4), glycodeoxycholic acid-d4 (GDCA-d4), and ursodeoxycholic acid-d4 (UDCA-d4) were the products of Cambridge Isotope Laboratories Inc (Tewksbury, MA, USA). LC-MS grade methanol, acetonitrile and formic acid were the products of Fisher Scientific. Other materials were obtained from Shanghai Anpel Laboratory Technologies (Shanghai, China). Bile acids supplementary products were supplied by Shandong Longchang Animal Health Care Co., Ltd., Dezhou, China (8.0% hyocholic acid (HCA), 70.9% hyodeoxycholic acid (HDCA), and 20.2% CDCA).

The ultra-high performance liquid chromatography-tandem mass spectrometry (UHPLC-MS/MS) utilized in the project was an Agilent 1290 Infinity II UHPLC coupled to an Agilent 6470A TripleQQQ (TQQQ) and AB SCIEX TripleTOF6600 (QTOF). The UPLC BEH C_18_ column (100 mm × 2.1 mm, 1.7 μm) (Waters, Milford, CT, USA) C18-Aq GracePure ^TM^ (500 mg/3 mL), was the product of Grace Davison Discovery Sciences ^TM^ (Waukegan Rd, IL, USA). The refrigerated centrifuge, Type 5430R, was bought from Eppendorf Inc, Germany. The Tissue Gnostics Fluorescence Imaging System was purchased from TissueGnostics (Vienna, Austria).

### 2.2. Bile and Plasma Sampling

As described in Yao et al.’s study [30], high plant (Cottonseed concentrate protein) protein diet (18% soybean meal and 18% cottonseed protein concentrated) (HP) and HP added 600 mg/kg BAs (HP+BAs) (HP+BAs) (Table 2) for common carp, respectively, were used. The fishes were fed to apparent satiation four times daily (8: 00, 11:00, 14:00 17:00) for 11 weeks in laboratory conditions to the re-circulating system. Six replicates were assigned to HP and HP+BAs groups, respectively, and every replicate distributed 30 fishes. Subsequently, the plasma, liver, and bile of stochastic twelve fishes from each group (two fishes from each replicate) were collected after obtaining an empty stomach for 24 h and stored at −80 °C for analysis. The body weight, hepatopancreas weight, and hepatopancreas intuitive phenotype of each fish were recorded in detail.

### 2.3. Plasma Biochemical Parameters

Plasma ALT (alanine aminotransferase), AST (aspartate aminotransferase), glucose, and total cholesterol (TC) were measured by Reagent kits (Nanjing Jiancheng Co., Nanjing, China) following the given protocols.

### 2.4. Histopathological Detections of Hepatopancreas Tissues

**Figure 1:**
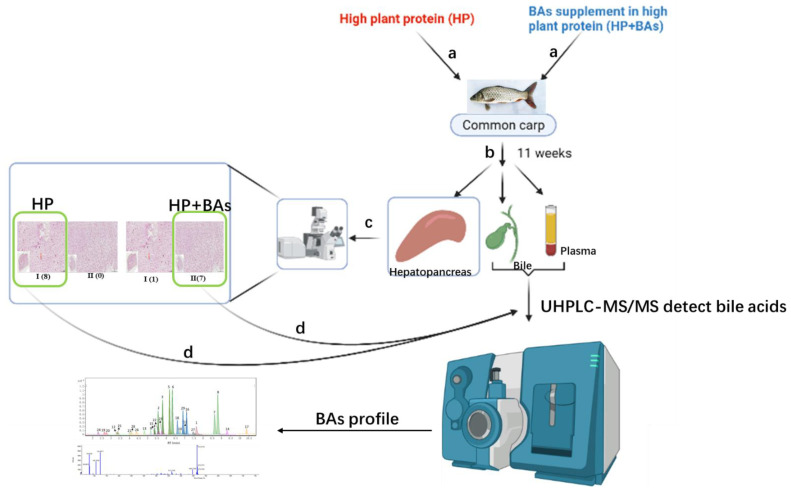
The workflow of the effect in bile acid supplement to high plant protein diet on common carp bile acid profile and hepatopancreas health. Arrows a: Common carp fed with HP and HP+BAs 11 weeks, respectively. Arrows b: Collect hepatopancreas, gallbladder, and plasma. Arrows c: Histopathological detections of hepatopancreas tissues. Arrows d: BAs analysis was performed on the bile and plasma corresponding to phenotype I of the hepatopancreas in the HP group (*n* = 8), the gallbladder and plasma corresponding to phenotype II of HP+BAs were treated in the same way (*n* = 7).

The hepatopancreas tissue fixation, dehydration, embedding, hematoxylin and eosin (H&E) and periodic acid Schiff (PAS) staining procedures were conducted as described by Yu et al. [32]. Then, the pictures were visualized using TissueGnostics Fluorescence Imaging System (TissueGnostics, Vienna, Austria) and the glycogen granules and effective nucleus analyzed by the StrataQuest Analysis Software (TissueGnostics, Vienna, Austria). BAs were extracted and analyzed for the corresponding plasma and bile samples with obvious hepatopancreas damage observed in HP group and no obvious abnormalities in the hepatopancreas observed in HP+BAs group. The graph abstract is shown in Figure 1.

### 2.5. Bile Acids Quantitative Analysis

Plasma and bile samples were prepared following the previous report [33]. The eluted substances of UHPLC-TQqQ-MS/MS were ionized in an electrospray ionization source in negative mode (ESI^−^). Both temperatures of ESI^−^ source drying gas and sheath gas were 300 °C. The flow rate of ESI- source drying gas and sheath gas were 5 and 11 L/min, respectively. The pressure of the nebulizer was 45 psi, and capillary voltage was 4000 V. The dynamic multiple reaction monitoring (dMRM) was used to acquire data in optimized MRM transition (precursor -> product), fragment, and collision energy (CE) as Table 1. The total scan time per cycle was 300 ms. Chromatographic separation was operated on a UPLC BEH C_18_ column (100 mm × 2.1 mm, 1.7 μm). The column temperature was 40 °C, and the flow rate was 0.45 mL/min. The mobile phase consisted of water in 0.1% formic acid (A) and acetonitrile in 0.1% formic acid (B). The chromatographic separation was conducted by a gradient elution program as follows: 0.5 min, 15% B; 1 min, 25% B; 3min, 25% B; 5 min, 34% B; 8 min, 40% B; 9 min, 52% B; 10.2 min, 58% B; 10.21 min, 100% B; 11.2 min, 100% B; 11.21 min, 15% B; 12.5 min, 15% B. The gradient elution was applied and MS detection proceeded in negative mode. Standards for all BAs were used to identify the different BA metabolites detected by UHPLC-MS/MS. The Agilent Mass Hunter software (version B.08.00) was used to control instruments and acquire data. The raw data were processed by Agilent Mass Hunter Workstation Software (version B.08.00) by using the default parameters and assisting manual inspection to ensure the qualitative and quantitative accuracies of each compound. The peak areas of target compounds were integrated and output for quantitative calculation.

### 2.6. TβMCA and TωMCA Qualitative Analysis

T*β*MCA and T*ω*MCA were qualified by UHPLC (Agilent 1290)-Q-TOF (AB SCIEX| 6600)-MS/MS with an ESI source. The main parameters of ESI-MS/MS were as follows: declustering potential (DP): −100 v, collision energy (CE): −60 v, ion source gas1 (GS1): 50 arb, ion source gas2 (GS2): 60 arb, curtain gas (CUR):30 arb, temperature: 600 °C.

Chromatographic separation was operated as 2.4. A mass range of *m*/*z* 50 to 1000 was acquired. PeakView 2.1 Software of AB SCIEX was used to analyze the ion fragment information of T*β*MCA and T*ω*MCA standards and samples.

### 2.7. Statistical Analyses

Independent-samples *t*-test of variance by the software SPSS Statistics 20 was used to analyze all data. Homogeneity test of variance (F-test) was also performed for the data between the two groups; log transformation analysis was executed on the data when variance was irregular. Data are presented as mean ± SEM. Statistically significant results are indicated by asterisks (*, *p* < 0.05; **, *p* < 0.01; ***, *p* < 0.001). Graphics were drawn using GraphPad Prism 8.0 (GraphPad Software Inc. USA). Hepatosomatic index (HSI) was calculated by the formula of weight of the hepatopancreas (g)/body weight (g)∗100%. All BAs’ unit conversion was calculated from ng/mL to mM, and we summed individual BA concentration as total BA concentration (TBA). The average concentration of individual BAs and the total BAs’ concentration were normalized to calculate the ratio of individual BAs to the total BAs.

## 3. Results

### 3.1. Growth Performance

**Figure 2:**
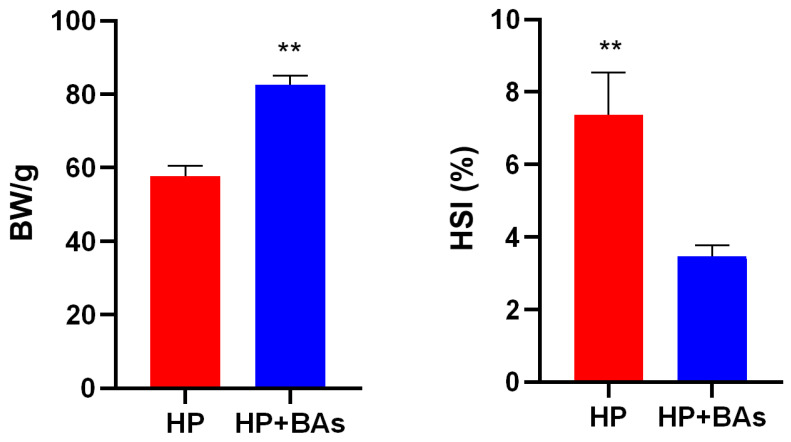
Supplement bile acids to high plant protein feed improved the growth performance and reduced the hepatosomatic index (HSI) in common carp (*Cyprinus carpio*). (Statistically significant results are indicated by asterisks (**, *p* < 0.01), *n* = 7.

Compared with the HP group, the growth performance of the HP+BAs group improved significantly (*p* < 0.01) (Figure 2), the final body weight (BW) increased significantly (*p* < 0.01), and HSI significantly decreased. The mean of HSI in HP and HP+BAs groups were 7% and 3%, respectively.

### 3.2. Hepatopancreas Histopathological Examination

**Figure 3:**
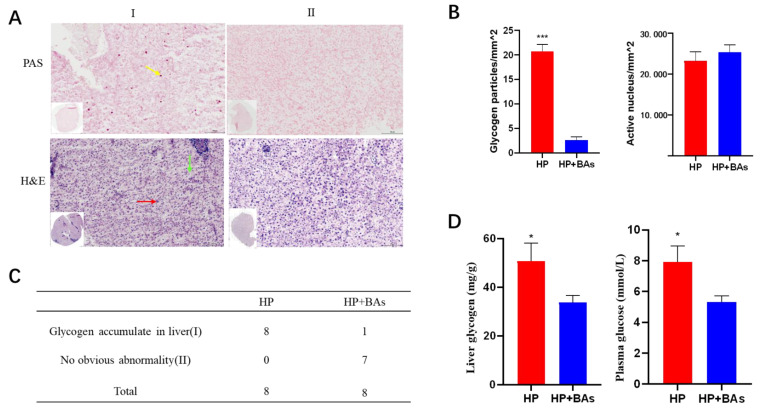
Supplement bile acids to high plant protein feed reduced carp hepatopancreas histological lesions (Statistically significant results were indicated by asterisks (*, *p* < 0.05; ***, *p* < 0.001)): (**A**) PAS and H&E staining of liver sections with bar = 100 μm, intracellular accumulation of glycogen (marked with yellow arrow), deformed cells (marked with green arrow) and Nuclear gathered (marked with yellow arrow) were clearly observed in the damaged hepatopancreas. (**B**) Quantification of glycogenosome and active nucleus, the number of hepatopancreas glycogenosome in the HP+BAs group carp was significantly lower than that in the HP group. (**C**) The phenotype of hepatopancreas histopathological examination in HP group and HP+BAs group. (**D**) Supplement BAs to high plant protein feed reduced hepatopancreas glycogen and plasma glucose (Statistically significant results are indicated by asterisks (*, *p* < 0.05; ***, *p* < 0.001), *n* = 7).

Fish hepatopancreas sections were examined after PAS staining and H & E staining, and eight samples were selected to be observed and quantified the glycogen granules and effective nucleus in each group. Two typical phenotypes are shown in Figure 3A. Phenotypes I: Hepatopancreas have obvious glycogen accumulation, vacuolization, blurred cell membrane boundaries, and cell nuclei aggregation. Phenotypes II: No significant accumulation of glycogen, and cell morphology showed no obvious abnormality, and effective nuclei also increased. Glycogen granules in the HP group were significant more than HP+BAs group (*p* < 0.001), while the HP+BAs group has a more active nucleus (Figure 3B).

### 3.3. Plasma Biochemical Parameters and Hepatic Glycogen

Plasma biochemical parameters of ALT, AST, and TC are listed in Table 3. ALT and AST in the HP group were apparently higher than HP+BAs (*p* < 0.05). Supplement BAs to a high plant protein diet not affected the content of TC in plasma. Plasma glucose and liver glycogen in the HP+BAs group were significantly lower (*p* < 0.05) than those in the HP group (Figure 3D).

### 3.4. Bile Acids Profile of in Common Carp Bile and Plasma

**Figure 4:**
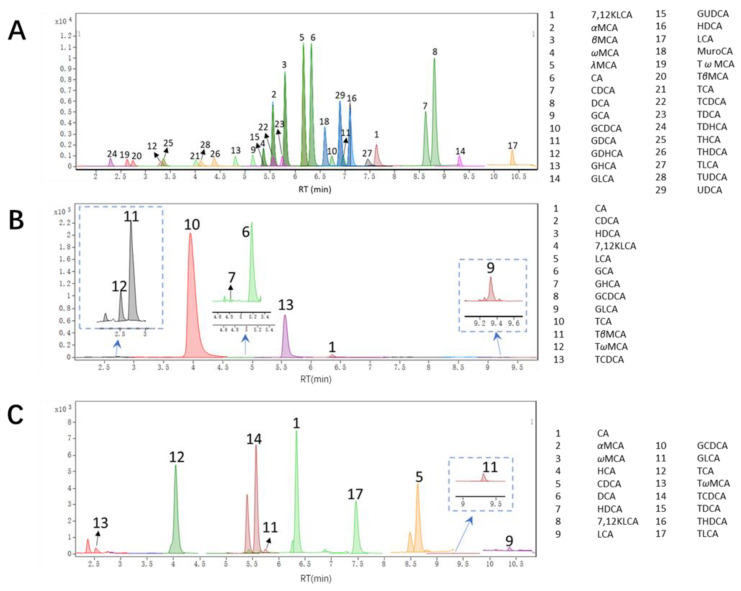
Bile acids Extracted Ion Chromatogram (EIC) in sample and standard; the peak times between bile acids do not interfere with each other. (**A**) is standard EIC, (**B**) is for bile sample, and (**C**) for plasma sample.

**Figure 5:**
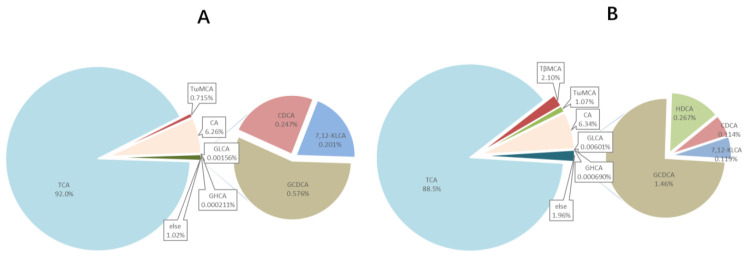
Bile acids species composition in bile, (**A**,**B**) for HP and HP+BAs, respectively (*n* = 7). TCA is the major BA of carp. Compared to HP group carp, the ratio of GCDCA, TβMCA, GLCA, and HDCA increased significantly in HP+BAs group, while TCA and CDCA decreased.

Ten compounds, including TCA, T*β*MCA, T*ω*MCA, CA, GLCA, GHCA, GCDCA, HDCA, CDCA, and 7,12-KLCA were found quantified in bile samples, whose EIC is shown in Figure 4. BA profiles of common carp bile are summarized in Table 4 and Figure 5, which suggested that TCA was the main bile acids in common carp, followed by CA which accounted for 88–92% and 6–7% respectively; CA ranged from 140–170 μM; however, no TCDCA existed.

Moreover, eight BAs, including TCDCA, CDCA, CA, LCA, HDCA, GLCA, GCDCA, and DCA were detected and quantified from the plasma samples. Table 5 shows the detailed BAs in bile and plasma.

**Figure 6:**
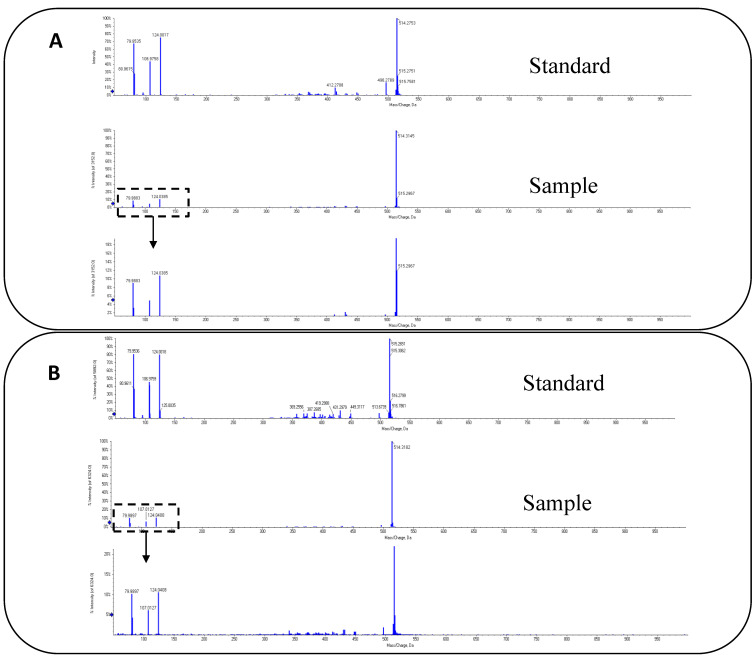
Tandem mass spectrometry (MS/MS) of TβMCA and T*ω*MCA in sample and standard. (**A**) for T*β*MCA, (**B**) for T*ω*MCA. The RT and abundance ratio of MS/MS of TβMCA and TωMCA standard were 2.79 min and 2.37 min, *m*/*z* 124.0017:106.9758:80.9615:79.9523 = 6:3:2:4 and *m*/*z* 124.0018: 106.9759:80.9611:79.9536 = 40:19:13:40, respectively, which in sample was consistent.

This was the first time that MCA was found to exist in fish. Thus, this was confirmed at this point. The confirmations of MCA detected were verified by the retention time (RT) and the abundance ratio of MS/MS. The RT and abundance ratio of MS/MS of T*β*MCA and T*ω*MCA standard were 2.79 min and 2.37 min, *m*/*z* 124.0017:106.9758:80.9615:79.9523 = 6:3:2:4, and *m*/*z* 124.0018:106.9759:80.9611:79.9536 = 40:19:13:40, respectively, which in the sample was consistent (Figure 6).

### 3.5. Supplement BAs to High Plant Protein Feed Altered the BA profile

**Figure 7:**
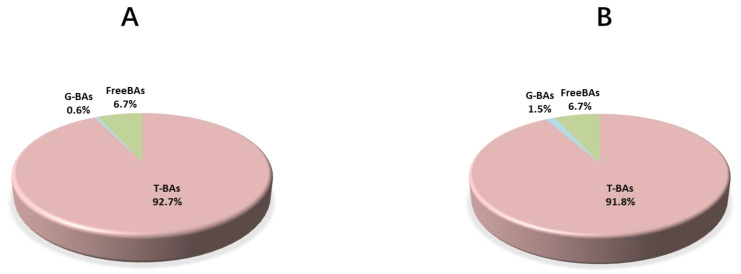
Supplement bile acids to a high plant protein diet increase the proportion of Glycine-conjugation bile acids significantly, while Tauro-conjugation bile acids decreased unremarkably in carp bile, (**A**,**B**) for HP and HP+BAs, respectively (*n* = 7).

The ratio of GCDCA, T*β*MCA, GLCA, and HDCA increased significantly in the HP+BAs group, while TCA and CDCA decreased (Figure 6). An increase of BA diversity in the HP+BAs group could be observed in both bile and plasma. Supplementary BAs to a high plant protein diet increased the proportion of G-BAs in bile, which accounted for 0.6% and 1.5% in the HP group and HP+BAs group, respectively (Figure 7).

## 4. Discussion

### 4.1. BA Profile Changes Caused by Supplements of BAs

TCA was the main BA in the common carp bile, which was consistent with the results of previously reported studies about the BA profile of fish (angelfish (*Pterophyllumeimekei*)) bile [24]. In this study, G-BAs such as GCDCA, GLCA, GHCA, and GCA were detected in fish. The BA family in animals is quite complex. Similar to the reports on the human [34], mice [35], and rainbow trout [36], we also found that the common carp can conjugate bile acid with glycine, but not only with taurine. In some early views, it was pointed out that animals, except for the mammals, conjugate their BAs exclusively with taurine [37,38], which led to the fact that less attention was paid to G-BAs when studying fish bile acids. The glycine conjugated bile acid in fish could be partially from fishmeal or other animal ingredients in the feed. We determined the bile acids of fish meal sample that we used in the present study, and the content of G-BAs is about 1.07 × 10^−7^ umol/mg. However, the fish meal in the experimental diets of this study only accounted for 10%, which made the bile acids level in the basal diet lower than the detection limit of LC-MS/MS. In the study of Staessen et al. (2021) [36], 27% fishmeal and 1.3% fish oil were used and about 1.1 × 10^−5^ umol/mg GBA were detected in the basal diet. In the present study, the dietary bile acid profile was composed with HCA (8.0%), HDCA (70.9%), and CDCA (20.2%), and the two experimental diets were designed with the same level of fishmeal. Hence, we can conclude that the increased GBA in the HP+Bas group should be majorly endogenous for the common carp. This is a report, for the first time, that bile acids can be conjugated with glycine in common carp, although it has been found that bile acids could hardly be combined with glycine in some fish species, such as Sea Lamprey [26] and lake charr [39]. In addition, compared with the HP group, the percentage of G-BA in the HP+BA group increased while the percentage of T-BA decreased; that is consistent with the finding that G-BA and T-BA have a mutual inhibition relationship in previous studies [40,41].

Supplementary BAs (mainly HCA, HDCA, CDCA) increased the contents of T*β*MCA, GLCA, GCDCA, and HDCA in the common carp bile; we suppose the following could account for this with the help of intestinal microorganisms. HCA transformed into *β*MCA by 6b-epimerization and further 7b-epimerization [42], then *β*MCA was reabsorbed back to hepatopancreas and combined with taurine into T*β*MCA, while HDCA was directly reabsorbed to the hepatopancreas. CDCA is dehydroxylated into LCA (based on KEGG secondary bile acid biosynthesis, map00121), which is partly excreted from the body, and part is reabsorbed to the liver and combined with glycine to form GLCA. The other part of CDCA is reabsorbed to the hepatopancreas to combine with glycine.

The discovery of T*β*MCA and T*ω*MCA in Common Carp was a breakthough since they were thought to be a rodent specific bile acid [42,43,44,45,46], and indeed, they were not found in birds and monogastric animal BA analysis [47,48], but have also been found in humans according to some reports [21,49]. Rodents branch off from fish in the evolutionary tree, and humans and rodents are on a small branch; we considered that MCA maybe a common species of bile acids that existed in fish, rodents, and primates. More work should be done in the future.

### 4.2. Supplement BAs Affected Common Carp BA Profile to Reduce Hepatopancreas Glycogen Accumulation and Alleviated Hepatopancreas Damage with a High Plant Protein Diet

Supplement BAs reduced liver glycogen accumulation and alleviated liver damage in common carp with a high plant protein diet. High plant protein could induce fish intestinal and liver damage that has been confirmed in the previous research through our laboratory [16,30,50,51]. It was reported a sturgeon intestinal obvious damage when it was fed a diet with more than 50% of plant protein content [50]. In this study, the proportion of plant protein was as high as 78%, and it has been confirmed by Yoa et al. [30] that this plant protein level causes the intestine of carp serious injury. The intestinal organ is the organ to digest and absorb nutrients, while damage and functional barriers would lead to nutritional metabolism disorders, especially proteins [52,53], thus affecting the synthesis of key enzymes of other nutrients. Therefore, in this study, the reason that a high-plant-protein diet caused hepatopancreas glycogen accumulation and damage is possible because the high-plant-protein diet injures the common carp’s intestines, leading to protein digestion and absorption disorders, and then resulting in a lack of phosphorylase (the key enzymes in liver glycogen decomposition) synthesis. In addition, disorders of glucose metabolism can stimulate cell inflammation and apoptosis, which cause liver damage [32,54]. It has been reported that supplementary BAs to high plant protein feed could alleviate intestinal and liver damage [30], and our results also confirmed that. The added BAs probably play a role as indicated in the following aspects to overcome the negative effects of high plant protein.

Firstly, the soysaponins of soybean meal may be the main cause of common carp intestinal damage [55]. BAs can be combined with non-starch polysaccharides and excreted from the body [56]. Saponins are composed of sapogenins and glycosyl, and a study has shown that soysaponins could increase the excretion of BAs [57]. This shows that soysaponins may have similar binding power to BAs as non-starch polysaccharides. Subsequently, BA supplements could be combined with saponins, thereby reducing the damage of saponins to the intestinal organ, and then improving protein digestion and absorption, reducing liver glycogen accumulation.

Secondly, common carp hepatopancreatic inflammation and glucose metabolism may be regulated by three purposes that were LCA, CDCA, and CA to activate liver TGR, increased liver glycine concentration and T*β*MCA inhibits intestinal FXR. TGR5 could be activated by some BAs, in which LCA is the most potent agonist for TGR5, DCA and the conjugations, CDCA and the conjugations, and CA and the conjugations activate TGR5 effectively simultaneously [58]. TGR5 plays an important role in anti-inflammatory activities and glucose metabolism [59]. TGR5 restrains the activated B cells (NF-κB) to control the proinflammatory factors secretion by the mediation of the interaction between IκBα and β-arrestin2 and thus exert anti-inflammatory effects [60,61,62]. Activating liver TGR5 could reduce blood glucose in mice with a high-fat diet [58]. That suggested the potential hypoglycemic function of TGR5. In the present study, plasma CDCA, CA, LCA, and GCDCA increased significantly in the HP+BAs group; with the assistance of enterohepatic circulation of Bas [63], they will enter the liver and activate TGR5, especially LCA, that cannot be synthesized directly in the liver while it can only be recovered from intestinal BAs by blood circulation, thus enhancing the anti-inflammatory ability of the body, regulating glucose metabolism and reducing blood glucose, and decreasing the liver inflammation [64]. The expression of the TGR5 gene in the liver increase after supplementation of BAs to HP has been confirmed by Yao et al. [30].

In addition, in the results of IDE et al. (1994), it can be found that the content of G-BAs in bile increases with the increase of liver glycine concentration [65]. Kupffer cells in an activated state could release a variety of inflammatory mediators and play a leading role when the liver is invaded, Glycine inactivates Kupffer cells and can protect the liver from inflammation [66]. T*β*MCA varied quite distinctly in bile between the HP group and HP+BAs group. T*β*MCA is a farnesoid X receptor (FXR) nuclear receptor antagonist [14]. FXR is a member of the nuclear receptor superfamily that is primarily expressed in the liver, kidney, and intestine [67]. In the FXR gene knockout mice, intestinal glucose absorption was delayed, together with blood glucose decreased [68]. T*β*MCA suppressed the enterohepatic FXR-FGF15 signaling and could affect glucose metabolism, reduce blood glucose, and treat diabetes [69]. Therefore, the increase of G-BAs and T*β*MCA in bile after the addition of BAs also plays a certain role in keeping the hepatopancreas from avoiding histological damage and the reduction of plasma glucose.

## 5. Conclusions

In summary, HP could induce glycogen accumulation in common carp hepatopancreas while supplemented BAs to HP could mitigate this symptom. BAs supplements in a high plant protein diet could change the BA profile of common carp, among them, plasma LCA, CDCA, and CA increased significantly, T*β*MCA and the proportion of G-BAs in bile increased significantly, which might play a leading role in it that reduced the accumulation of hepatopancreas glycogen and maintained hepatopancreas health. This study proceeded with an integrated bile acid profile determination by UHPLC-MS/MS to identify the effect of exogenous BAs supplementary on the endogenous BA profile and hepatopancreas health of common carp and discussed how the BAs supplementary is transformed in the body, providing a theoretical basis for the application of BAs products in fish and a data basis for revealing the mystery of fish BAs. In addition, this study has important significance for the development of aquaculture and has a potential contribution to expanding the strategic space of food security.

## Figures and Tables

**Table 1:** Parameters for quantification on BAs by UHPLC-MS/MS.

No.	Compounds	Retention Time	Transition	Fragmentor	CE	Linearity Range	Calibration Curves *	*R* ^2^	Internal Standard
		(min)	(*m*/*z*)	(V)	(eV)	(μg/mL)			
1	CA	6.339	407.3 -> 407.3	240	10	0.0025–5	y = 13.798x	0.9989	CA -d4
2	αMCA	5.570	407.3 -> 407.3	240	10	0.001–5	y = 100.08x	0.9992	βMCA -d5
3	ωMCA	5.384	407.3 -> 407.3	240	10	0.001–5	y = 65.329x	0.9982	βMCA -d5
4	HCA	6.178	407.3 -> 407.3	240	10	0.001–5	y = 11.074x	0.9994	βMCA -d5
5	CDCA	8.628	391.3 -> 391.3	230	10	0.001–5	y = 39.802x	0.9995	CDCA-d4
6	DCA	8.805	391.3 -> 391.3	230	10	0.001–5	y = 8.9922x	0.9998	DCA-d4
7	HDCA	7.110	391.3 -> 391.3	230	10	0.001–5	y = 8.6365x	0.9993	DCA-d4
8	7,12KLCA	7.641	389.3 -> 389.3	225	10	0.005–0.5	y = 52.86x	0.9934	LCA-d4
9	LCA	10.367	375.3 -> 375.3	235	10	0.005–0.5	y = 17.63x	0.9972	LCA-d4
10	GCA	5.169	464.3 -> 74.0	230	46	0.001–5	y = 44.935x	0.9926	GCA-d4
11	GHCA	4.823	464.3 -> 74.0	230	46	0.001–5	y = 118.29x	0.9974	GCDCA-d4
12	GCDCA	6.744	448.3 -> 74.0	220	40	0.001–5	y = 27.884x	0.9930	GCDCA-d4
13	GLCA	9.304	432.3 -> 74.0	225	36	0.001–5	y = 17.814x	0.9996	GLCA-d4
14	TCA	4.059	514.3 -> 79.9	300	77	0.001–5	y = 32.025x + 0.2914	0.9989	TCA-d5
15	TβMCA	2.787	514.3 -> 79.9	300	77	0.005–5	y = 28.963x − 0.0743	0.9966	TβMCA -d4
16	TωMCA	2.371	514.3 -> 79.9	300	77	0.005–5	y = 28.963x − 0.0743	0.9966	TβMCA -d4
17	TCDCA	5.572	498.3 -> 79.9	280	80	0.001–5	y = 51.689x + 0.0782	0.9999	TUDCA-d5
18	TDCA	5.753	498.3 -> 79.9	280	80	0.001–5	y = 191.74x	0.9950	TDCA-d5
19	THDCA	4.412	498.3 -> 79.9	280	80	0.001–5	y = 48.675x − 0.0104	0.9999	TUDCA-d5
20	TLCA	7.463	482.3 -> 79.9	290	80	0.001–0.5	y = 9.4573x	0.9931	LCA-d4
21	βMCA	5.812	407.3 -> 407.3	240	10	0.001–5	y = 83.77x + 0.1057	0.9995	βMCA -d5
22	UDCA	6.913	391.3 -> 391.3	230	10	0.001–5	y = 61.645x + 1.3425	0.9991	UDCA-d4
23	MuroCA	6.622	391.3 -> 391.3	230	10	0.0025–5	y = 1.2135x + 0.0362	0.9993	DCA-d4
24	GDCA	6.976	448.3 -> 74.0	220	40	0.001–5	y = 7.6437x + 0.0541	0.9997	GDCA-d4
25	GUDCA	5.387	448.3 -> 74.0	220	40	0.005–5	y = 130.8x − 5.5375	0.9982	GCDCA-d4
26	GDHCA	3.354	458.2 -> 74.0	205	36	0.001–5	y = 88.496x + 4.2369	0.9973	GCDCA-d4
27	TαMCA	2.657	514.3 -> 79.9	300	77	0.001–5	y = 27.164x − 0.0574	0.9977	TβMCA -d4
28	THCA	3.405	514.3 -> 79.9	300	77	0.0025–5	y = 23.806x + 0.1518	0.9994	TDCA-d5
29	TUDCA	4.151	498.3 -> 79.9	280	80	0.001–5	y = 35.379x − 0.1025	0.9999	TUDCA-d5
30	TDHCA	2.314	508.3 -> 79.9	285	72	0.001–5	y = 15.83x + 0.1023	0.9997	TUDCA-d5

* y, the integral peak area ratio between standard and IS (internal standard); x, concentration in the detected samples or standard curves samples.

**Table 2:** Experimental diets’ formula and composition of HP and HP+BAs groups (air-dry basis, %) (The content of this table has been published [30]).

Feed Formulation	HP	HP+BAs
Fish meal ^a^	10.00	10.00
Soybean meal	18.00	18.00
Cottonseed protein concentrated	18.00	18.00
Tapioca flour	5.00	5.00
Wheat flour	39.80	39.74
Soy oil	4.00	4.00
Vitamin and mineral premix ^b^	4.10	4.10
Lecithin oi	1.00	1.00
DL-me	0.10	0.10
Total	100	100
Bile Acid (mg/kg) ^c^	0	600
Analyzed nutrients compositions		
Moisture	8.24	9.11
Crude protein	30.47	29.15
Crude lipid	7.53	7.47
Crude Ash	7.25	7.31
Gross energy (MJ/Kg)	18.28	18.20

^a^ Fish meal: Shandong Chishan Fishmeal Factory, Shandong, China; Soybean meal: Yihai Kerry Investment Co. Ltd., China; CPC: Xinjiang Jinlan Plant Protein Co. Ltd., China. ^b^ Vitamin premix (mg·kg-1 diets): Vitamin A 28; Vitamin B1 12; Vitamin B212; Vitamin B6 16; Vitamin B12 0.2; Vitamin E 300; Vitamin K3 20, Vitamin D 14; Niacinamide 80; Vitamin C 600; Calciumpantothenate 100; Biotin 0.4; Folicacid 3; Corn protein powder 314.4. Mineral premix (mg·kg^−1^ diets): FeSO_4_·H_2_O 300; ZnSO_4_·H2O 300; MnSO4·H2O 100; Na_2_SeO_3_ 10; CoCl_2_·6H_2_O (10% Co) 2.5; Kl 80; Zeolite 1307.5; MgSO_4_ 500. ^c^ Bile acids: supplied by Shandong Longchang Animal Health Care Co. Ltd. (Dezhou, China), with 8.0%HCA, 70.9% HDCA, and 20.2% CDCA. Bile acids were added and well mixed in premix at levels of 0 and 600 mg/kg, respectively.

**Table 3:** Plasma biochemical parameters. The concentrations of AST and ALT in the HP+BAs group was significantly lower than in the HP group. * Data are shown as mean ± SEM (*n* = 7). Statistically significant results are indicated by asterisks (*, *p* < 0.05).

	HP	HP+BAs
AST(U/L)	103 ± 11.8 *	62.0 ± 5.84
ALT(U/L)	73.7 ± 9.97 *	46.5 ± 5.50
TC (mmol/L)	5.2 ± 0.308	5.3 ± 0.345

**Table 4:** Bile acid profile in bile of common carp in HP and HP+BAs groups (μmol/L), T*β*MCA, GLCA, GHCA, and GCDCA increased observably in the HP+BAs group carp bile. * Data are shown as mean ± SEM (*n* = 7). Statistically significant results are indicated by asterisks (**, *p* < 0.01; ***, *p* < 0.001).

	BAs in Bile	HP	HP+BAs
Free BAs	CA	162.0 ± 51.0	149.7 ± 30.0
	HDCA	ND	6.3 ± 2.4
	LCA	ND	3.8 × 10^−2^ ± 7.1 × 10^−3^
	DCA	7.4 × 10^−4^ ± 1.9 × 10^−4^	ND
	CDCA	6.4 ± 1.7	2.7 ± 0.7
	7,12-KLCA	5.2 ± 1.0	2.8 ± 0.2
T-BAs	TCA	2380.6 ± 356.4	2091.1 ± 262.4
	TCDCA	ND	ND
	T*β*MCA	ND	49.6 ± 5.4 **
	T*ω*MCA	18.5 ± 3.7	25.3 ± 2.5
G-BAs	GCA	ND	ND
	GLCA	4.0 × 10^−2^ ± 1.6 × 10^−3^	1.4 × 10^−1^ ± 1.2 × 10^−2^ **
	GHCA	5.5 × 10^−3^ ± 2.8 × 10^−4^	1.6 × 10^−2^ ± 2.7 × 10^−3^ **
	GCDCA	14.9 ± 0.9	34.5 ± 2.6 ***
TBA		2561.7 ± 346.0	2337.7 ± 286.9

**Table 5:** Bile acid profile in plasma of common carp in HP and HP+BAs groups (μmol/L), CA, CDCA, LCA, and GCDCA increased observably in the HP+BAs group carp plasma. * Data are shown as mean ± SEM (*n* = 7). Statistically significant results are indicated by asterisks (*, *p* < 0.05).

	BAs in Plasma	HP	HP+BAs
Free BAs	CA	ND	5.7 × 10^−3^ ± 2.0 × 10^−3^ *
	HDCA	3.5 × 10^−3^ ± 2.1 × 10^−3^	5.0 × 10^−3^ ± 1.1 × 10^−3^
	CDCA	ND	2.0 × 10^−3^ ± 3.5 × 10^−4^ *
	7,12-KLCA	ND	ND
	LCA	ND	3.8 × 10^−2^ ± 7.06 × 10^−3^ *
T-BAs	TCA	ND	ND
	TCDCA	1.1 × 10^−1^ ± 1.3 × 10^−2^	2.3 × 10^−1^ ± 7.4 × 10^−2^
	T*β*MCA	ND	ND
	T*ω*MCA	ND	ND
G-BAs	GCA	ND	ND
	GLCA	8.5 × 10^−5^ ± 3.2 × 10^−5^	1.0 × 10^−4^ ± 3.10 × 10^−8^
	GHCA	ND	ND
	GCDCA	ND	3.7 × 10^−3^ ± 5.2 × 10^−4^ *
TBA		1.3 × 10^−1^ ± 1.9 × 10^−2^	3.2 × 10^−1^ ± 9.4 × 10^−2^
